# Newborn of mothers affected by autoimmune thyroiditis: the importance of thyroid function monitoring in the first months of life

**DOI:** 10.1186/1824-7288-36-24

**Published:** 2010-03-10

**Authors:** Rosanna Rovelli, Maria Cristina Vigone, Chiara Giovanettoni, Arianna Passoni, Ludovica Maina, Andrea Corrias, Carlo Corbetta, Fabio Mosca, Giuseppe Chiumello, Giovanna Weber

**Affiliations:** 1 Department of Pediatrics, San Raffaele Scientific Institute, Vita - Salute San Raffaele University, Milan, Italy; 2 Regina Margherita Hospital, Department of Pediatric Endocrinology, Turin, Italy; 3 Laboratory for Neonatal Screening, Buzzi Children Hospital, Milan, Italy; 4 NICU - Fondazione IRCCS, Ospedale Maggiore Policlinico, Mangiagalli e Regina Elena, University of Milan, Italy

## Abstract

**Background:**

evaluation of thyroid function in neonates born from mothers affected by autoimmune thyroiditis in order to define if a precise follow-up is necessary for these children. The influence of maternal thyroid peroxidase antibody (TPOAb) and L-thyroxine therapy during pregnancy on neonatal thyroid function was also investigated.

**Methods:**

129 neonates were tested for thyroid function by measurement of free thyroxine (FT4) and thyroid stimulating hormone (TSH) in 3^th ^day, 15^th ^day and at one month of life. TPOAb were measured in all patients; periodical control of thyroid function were performed until 6 months of life if Ab were positive. Data concerning etiology of maternal hypothyroidism and maternal replacement therapy with L-thyroxine during pregnancy were retrospectively collected.

**Results:**

28% neonates showed at least a mild increase of TSH value at the different determinations. In the majority of them, a spontaneous completely normalisation of TSH value was observed within the first month life. L-thyroxine replacement therapy was started in 3 neonates. TPOAb titer and maternal L-thyroxine replacement therapy were not related to alteration of thyroid hormone function in our study population.

**Conclusions:**

transient mild elevation of serum TSH above the normal reference value for age is frequently observed in the first month of life in infants born from mothers affected by autoimmune thyroiditis. Persistent hyperthyrotropinemia requiring replacement therapy is observed in 2.2% of these neonates. According to our experience, follow-up is recommended in these newborns; the most accurate and not invasive way to carefully monitor these infants after neonatal screening for CH seems to be serum-testing TSH between 2^nd^and 4^th ^week of life.

## Background

During pregnancy there are many physiological changes of maternal thyroid function. Hypothyroidism has been reported in around 2.5% of otherwise normal pregnancies [1,2] Hypothyroidism, if undiagnosed and untreated, could cause not only obstetric complications, such as hypertension, placental abruption, preterm delivery, low birth weight, but also exposes fetus to low thyroid hormone levels. The lack of maternal thyroid hormone during early pregnancy might have some irreversible effects on fetal development [3]. It is now clear that there is a close relationship between maternal thyroid deficiencies and the neuropsychological development of her child. Several studies demonstrated that maternal hypothyroidism was associated with impaired psychomotor and intellectual development of the child [4-8]. In order to reproduce the physiologic changes of thyroid function during gestation, women with known hypothyroidism and receiving L-thyroxine before pregnancy should increase their dosage by 30% to 60% during pregnancy [9,10].

The most common cause of hypothyroidism during gestation is autoimmune thyroiditis, which is characterised by the presence of specific thyroid autoantibodies. The presence of thyroid peroxidase antibodies (TPOAb) is frequent associated to thyroiditis in women of childbearing age; TPOAb have been found in 10% of women during or shortly after pregnancy [11]. Dussault et al reported that there is no correlation between the presence of maternal antimicrosomal antibodies and congenital hypothyroidism[12]; a further study conducted in Quebec in 1999 demonstrated an increased prevalence of transient congenital hypothyroidism associated with maternal autoimmune thyroid disease, particularly antimicrosomal autoantibodies were found in 77% of mothers of infants with transient congenital hypothyroidism[13]. Furthermore, the presence of maternal TPOAb with normal maternal thyroid function has been described in association with impaired neuropsychological development during early childhood [14].

Autoimmune thyroiditis characterised by the presence of TSH receptor antibodies is a less frequent disorder and its incidence in pregnancy is not well known. Maternal-to-fetal transfer of TSH receptor-blocking antibodies can lead to a rare condition of transient congenital hypothyroidism. The incidence of this disorder in North America is 1 in 180.000 normal infants, or approximately 2% of babies with congenital hypothyroidism[15,16]; Prospective studies about thyroid function in neonates born from mothers affected by autoimmune thyroiditis are not available in literature; in particular is not yet known if maternal thyroiditis could influence thyroid hormone levels of the neonate in the first period of life. Even if cases of CH related to the presence of maternal TSH-receptor antibodies are well documented in literature as noted above, data about the exact impact of maternal TPO-antibodies on neonatal thyroid function are not available.

Considering the high incidence of TPOAb in pregnant women, we aimed to establish if they could interfere with neonatal thyroid function in order to standardize an appropriate follow-up for these children. We also evaluated the relationship between maternal replacement therapy adjustment during pregnancy and newborn thyroid function status during the first months of life.

## Methods

Between April 2003 and May 2006, 129 neonates born from mothers affected by autoimmune thyroiditis were referred to Endocrinologic Neonatal Unit of Our Centre. A longitudinal prospective study was conducted on this population. Exclusion criteria for the study were: preterm delivery, perinatal asphyxia and congenital disease.

Data at birth including gestational age, delivery, Apgar score, weight, lenght, head circumference, were recorded.

Newborn screening for CH was performed and recorded in all neonates included in the present study. Regional screening program is conducted on filter paper blood samples in 3-4 day of life by measurement of TSH, with b-TSH cutoff of 10 mU/L.

TSH and FT4 on serum samples were measured in 3^th ^- 4^th ^day of life and repeated at about 15 days of life and at 30 days of life; TPOAb were determined concurrent with the sampling performed in 15^th ^day. TSH and FT4 measurement was repeated at 3 months and at 6 months of life if TPOAb were positive, or more closely if thyroid hormone levels were not in the reference range for age. TPOAb titer was reapeted at 6 months in order to evaluate if the wash-out was completed. If antibodies were negative and hormone levels resulted in the reference range for age, follow up was stopped at one month of life.

L-thyroxine replacement therapy was started if TSH was persistently higher or FT4 lower than the reference range for age according to Ranke's reference range[17]. Neonates were investigated about the aetiology of CH by performing thyroid scan, dosage of anti TSH receptor antibodies and urinary iodine before treatment. Neuropsychological evaluation using Griffith's scale was performed in the neonates who underwent replacement therapy between six months and one year of life.

Clinical and auxological examinations were performed by the Pediatrician at one month of life in all neonates and at 3 and at 6 months in children whose follow-up continued for positive TPOAb or for thyroid hormone levels alteration; the presence of symptoms and signs of dysthyroidism was considered.

The diagnosis of maternal hypothyroidism was usually performed by the referring physician before pregnancy and by the ginecologist during gestation. Management of these woman during pregnancy was conducted by their endocrinologists or gynecologists/obstetricians. Thyroid function was periodically measured during gestation and L-thyroxine replacement therapy was started or adjusted in order to maintain hormone levels in the normal referring range. Informations about pregnancy were retrospectively collected from maternal anamnestic history; at the first consultation, women were asked about aetiology of hypothyroidism, about their hormonal levels and L-thyroxine therapy management during pregnancy. None of the Hashimoto's affected women referred to have low serum FT4 levels during pregnancy.

Written informed consent was obtained from patients for publication of this manuscript.

Collected data were expressed as median and range. Results were analysed using Kruskall-Wallis test. Statistics were performed using Sygma Stat.

## Results

129 neonates (61 females, 68 males) born from mothers affected by autoimmune thyroiditis were enrolled in the present study. All women referred to be biochemically euthyroid throughout pregnancy; the dose of L-thyroxine was increased during 88 pregnancies, 41 patients mantained the dosage before pregnancy.

All neonates were born at term, at 39 median gestational week (range: 36 - 41+4 g.w.). Caesaren section was performed in 41 cases.

Median birth weight was 3292.5 gr (range: 2100-4400 g), median lenght was 50 cm (range: 45-56 cm), median head circumference was 34 cm (range: 31-44 cm); Apgar score was ≥ 7 at one minute and at 5 minutes for all children.

Neonatal screening for CH was negative in all patients in our population.

FT4 value was in the normal reference range for age at any determination in all neonates[17].

36/129 (28%) neonates showed at least a TSH value higher than the reference range for age at different blood testing during our follow-up. 27/36 (75%) of them were born by spontaneous delivery; only 9 (25%) of them were born by cesarean section.

In 3^th ^- 4^th ^day of life 30/129 neonates showed pathologic TSH value (median value: 11.86 mU/L, range 8,54 - 35.37 mU/L) considering the normal reference range for age, with definitive spontaneous normalisation in 28 cases of them (93.3%) within 15 days of life (25 patients) and one month of life (3 patients). 2 patients started replacement therapy with L-thyroxine at about one month of life for persistent mild elevation of TSH. (Patients n. 1, 2 - Table 1)

**Table 1:** TSH value* at different determination during the first month of life in neonates who started replacement therapy

Patients	TSH in 3^th ^day	TSH in 15^th ^- 20^th ^day	TSH at about 1 month
**n. 1**	26.40 mU/L	10.95 mU/L	10.52 mU/L Started therapy
**n. 2**	9.30 mU/L	8.18 mU/L	13.62 mU/L Started therapy
**n. 3**	N	11.24 mU/L (one week later:11) Started therapy	

* TSH normal reference value[17]:

< 1 month: 0,5-8,7 mU/ml

1 month - 1 year: 0,4-6,3 mU/ml

In 15^th ^day of life, 2/129 patients showed transient mild increment of TSH value (9.86 mU/L in one case and 11.8 mU/L in the other) which normalized at subsequent controls. One neonate whose TSH value was in the normal reference range at the first determination in 3^th ^day of life, showed TSH alteration (11.24 mU/L) in 15^th ^day of life which was confirmed at a further control performed a few days later (TSH 11.00 mU/L). L-thyroxine replacement therapy was started at about 20 days of life in this case. (Patients n.3 in table 1)

3 other neonates showed transient isolated TSH elevation (6.5 - 10.0 - 10.1 mU/L) between one and three months of age, which was not confirmed thereafter so that therapy was not necessary.

TSH was normal at 6 months in non-treated children.

TPOAb were present in 73/129 neonates (59%) in 15^th ^day of life (normal range value 0-100 IU/ml). At 6 months of life autoantibodies were absent in all patients. Considering 36 newborns with at least an alteration of TSH, TPOAb were present in 17 (47%) of them.

As described above, replacement therapy with L-thyroxine was started in 3/129 patients (2.2%). Before starting therapy, neonates were investigated in order to establish the diagnosis of their hyperthyrotropinemia. All of them were born by spontaneous delivery. Normal thyroid gland in situ was present in all neonates at thyroid scan. TPOAb were present in two patients who started replacement therapy and were absent in the other; rTSH-Ab were absent in all neonates who started therapy. None of them showed elevated values of urinary iodine. After an appropriate discontinuation of therapy between one and two years of life, all three patients restore a normal thyroid function. Additionally their neuropsychological development, evaluated between six months and one year of life using Griffith's scale, resulted adequate (DQ 102-105).

We also evaluated the relation between maternal L-thyroxine replacement therapy during pregnancy and hormonal results of newborns. 88 neonates (68%) were born from mothers whose therapy was increased during gestation. Maternal therapy was adequately adjusted during pregnancy in 30/36 neonates (83%) who showed alteration of TSH.

All children showed normal auxological parameters during our follow-up; neither signs of dysthyroidism or medical problems were observed.

## Discussion

The results of the present study indicate that mild thyroid dysfunction characterized by TSH increase with normal FT4 level is frequently observed in the first month of life in neonates born from mothers affected by autoimmune thyroiditis. The majority of them undergoes to complete and spontaneous normalisation of TSH value. Persistent hyperthyrotropinemia requiring replacement therapy is demonstrated in 2.2% of these neonates. No alteration of thyroid function was observed after the first month of life in children who had positive TPOAb. The limit of this paper is the lack of a control population.

TSH increase in 3^th ^day of life is often transient; it could be attributable to development of the hypothalamic-pituitary-thyroid axis at birth. In our study population, complete normalisation of TSH value within 15-20 days of life was observed in 28/30 neonates (93.3%) who showed a mild increase in 3^th ^day of life. Neonatal screening programs for CH, which is routinely performed between 3-4 days of life in all newborns, provides a sufficient and available source for identification of early TSH increment. Interestingly, one neonate who resulted negative to the neonatal screening for CH and whose TSH was normal at the first plasma TSH sampling, showed TSH value above the normal reference range in 15^th ^day of life; TSH measurement was repeated and confirmed, so that replacement therapy was started at about one month of life. The only indication to blood testing this neonate for thyroid function was maternal autoimmune thyroiditis, which allowed to early identification of hyperthyrotropinemia. Our data suggest that follow-up of neonates born from mothers affected by autoimmune thyroiditis could be performed by neonatal screening performed on filter blood sample followed by measurement of TSH and FT4 on serum between 2^nd ^and 4^th ^weeks of life in order to early identify any possible alteration of thyroid hormones. Some screening programs routinely performed a second screening in high-risk patient groups [18]. So in these cases we suggest, as an alternative strategy, to repeat, between 2^nd ^and 4^th ^weeks of life, the measurement of TSH on filter paper.

In children that require therapy it would be helpful try to stop therapy to value the transitoriness of the disease and continue to monitor the child carefully and repeat the thyroid function tests at the slightest suspicion of recurrence of hypothyroid symptoms.

The presence of TPOAb in neonates with normal TSH and in those with TSH alteration was similar in our population. We didn't demonstrate any relation between TSH value and TPOAb presence/absence at any blood testing and in neonates who started therapy (TPOAb were present in 2 of them and negative in the other). Our data are in agreement with other studies [12], which showed the lack of influence of antibodies on thyroid function in the newborn infant, and confirms that TPOAb an TGAb apparently have no pathogenetic effect on fetal and neonatal hypothyroidism [19].

Children were investigated about rTSH-Ab titer and thyroid scan only if they showed persistent hyperthyrotropinemia in order to better define its etiology. rTSH-Ab titer was measured in children who started therapy; they were negative in all neonates who started replacement therapy, indicating no influence of maternal rTSH-Ab on neonatal thyroid function. Our data provide further evidence that rTSH-Ab dosage is not systematic recommended in infants born from mothers affected by autoimmune thyroiditis, even if it remains useful in all neonates who show persistent elevation of TSH. The scan was performed to exclude severe gland's hypoplasia, any disomogeneity or ectopic glands, which could represent a cause of thyroid function's alteration.

Considering the potential benefit of L-thyroxine dose adjustment in hypothyroid women during pregnancy in determining the neuropsychological development of the children [6,7] we evaluated maternal treatment during pregnancy. Studies conducted on mothers of congenitally hypothyroid infants showed that suppressed and elevated TSH concentrations were more frequent in the mothers of infant with congenital hypothyroidism. We considered if adequacy of therapy during pregnancy could influence neonatal thyroid function. The results of the present study indicate that adjustment of L-thyroxine treatment during pregnancy was not related with thyroid function of the newborns. Percentage of women who increased dosage of L-thyroxine was similar in neonates with TSH alteration and in neonates with normal TSH level during our follow-up; furthermore neonates who started therapy were born from mothers whose therapy has been increased during pregnancy. We didn't demonstrate a possible influence of maternal on perinatal thyroid hormone function; a longer follow-up is needed to evaluate the impact of L-thyroxine treatment on neuropsychological development of children.

## Conclusion

In conclusion our data show the utility of monitoring thyroid function in neonates born from mothers affected by autoimmune thyroiditis. Our results confirm the possibility of mild alteration of TSH in the first months of life, which could be difficulty diagnosed when signs or symptoms are absent.

After neonatal screening for CH which is routinely performed in all neonates by measurement of TSH or TSH/FT4, the most prudent policy would be FT4 and TSH serum testing between 2^nd ^and 4^th ^week of life in all neonates born from mothers affected by autoimmune thyroiditis; if hormone values resulted in the normal referring range follow-up could be stopped. In addition in children that require therapy it would be helpful try to stop therapy to value the transitoriness of the disease and continue to monitor the child carefully and repeat the thyroid function tests at the slightest suspicion of recurrence of hypothyroid symptoms. In order to obtain helpful information regarding etiology and prognosis in cases of persistent TSH abnormality we advice to measure TPOAb and/or rTSH-Ab and to perform thyroid scan.

Further study are needed to evaluate the neuropsychological outcome of this children. Collaboration with endocrinologist, gynecologist and neonatologist would be necessary for a correct approach of women affected by hypothyroidism during pregnancy and for management of the neonates during the first month of life.

## Competing interests

The authors declare that they have no competing interests.

## Authors' contributions

RR, MCV, CG, AP and LM were contributors in writing the manuscript. GW conceived of the study, and participated in its design and coordination and helped to draft the manuscript. All authors read and approved the final manuscript.
